# Bioprospecting Microbiome for Soil and Plant Health Management Amidst Huanglongbing Threat in Citrus: A Review

**DOI:** 10.3389/fpls.2022.858842

**Published:** 2022-04-26

**Authors:** Anoop Kumar Srivastava, Ashis Kumar Das, Prasanth Tej Kumar Jagannadham, Popy Bora, Firoz Ahmad Ansari, Ruchi Bhate

**Affiliations:** ^1^Indian Council of Agricultural Research (ICAR)-Central Citrus Research Institute, Nagpur, India; ^2^Department of Plant Pathology, Assam Agricultural University, Jorhat, India

**Keywords:** citrus, microbiome, soil health, plant health, Huanglongbing

## Abstract

Microorganisms have dynamic and complex interactions with their hosts. Diverse microbial communities residing near, on, and within the plants, called phytobiome, are an essential part of plant health and productivity. Exploiting citrus-associated microbiomes represents a scientific approach toward sustained and environment-friendly module of citrus production, though periodically exposed to several threats, with Huanglongbing (HLB) predominantly being most influential. Exploring the composition and function of the citrus microbiome, and possible microbial redesigning under HLB disease pressure has sparked renewed interest in recent times. A concise account of various achievements in understanding the citrus-associated microbiome, in various niche environments viz., rhizosphere, phyllosphere, endosphere, and core microbiota alongside their functional attributes has been thoroughly reviewed and presented. Efforts were also made to analyze the actual role of the citrus microbiome in soil fertility and resilience, interaction with and suppression of invading pathogens along with native microbial communities and their consequences thereupon. Despite the desired potential of the citrus microbiota to counter different pathogenic diseases, utilizing the citrus microbiome for beneficial applications at the field level is yet to be translated as a commercial product. We anticipate that advancement in multiomics technologies, high-throughput sequencing and culturing, genome editing tools, artificial intelligence, and microbial consortia will provide some exciting avenues for citrus microbiome research and microbial manipulation to improve the health and productivity of citrus plants.

## Introduction

Plants recruit a broad array of microbes surviving in different tiers of their habitat viz., rhizosphere, phyllosphere, and endosphere, predominantly represented by bacteria, archaea, fungi, actinomycetes, protists, and other living entities. These microbial communities constitute structural, as well as functional micro-communities, associated with various parts of the plants under natural environment, are collectively coined as plant microbiomes or phytobiome (Busby et al., 2017; de Souza et al., 2020; Pascale et al., 2020). These microbes either live internally known as endophytes or externally known as epiphytic or rhizospheric microbes (Trivedi et al., 2020). Plant microbiomes influence the plant’s functionality in multiple directions, including supply-chain of essential nutrients to plants, determining the soil fertility vis-à-vis plant health, stimulating plant growth, enhancing biotic and abiotic stress tolerance, sustaining plant health by competing against phytopathogens, induction of resistance, driving the emergence of multiple disease resistance systems through long eventful history of co-evolution of plant-microbiome association (Reinhold-Hurek et al., 2015; Vandenkoornhuyse et al., 2015; Lazcano et al., 2021). There are inevitable reasons that the plant microbiome is deliberated as an essential component of host-plant assemblage, known as a holobiont (Zhang et al., 2017).

**FIGURE 1:**
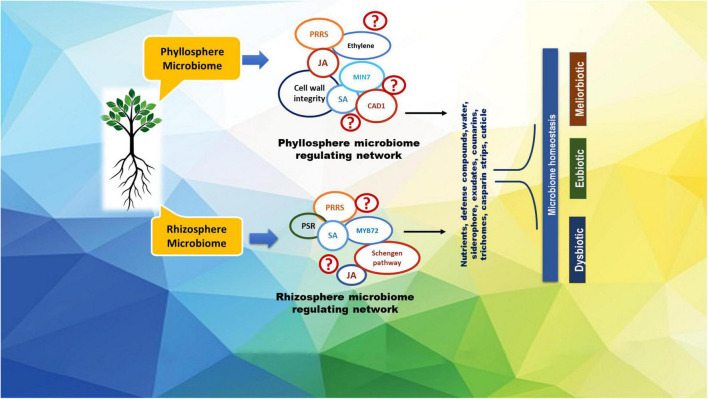
A representative roadmap of the regulation of citrus microbiome in plant homeostasis. Some of the known regulators of the plant host involved in microbiome homeostasis are depicted in the figure as CAD1, constitutively activated cell death 1; JA, jasmonic acid; MIN7, HOPM1-interactor 7; MYB72, MYB domain protein 72; PRRs, pattern recognition receptors; PSR, phosphate starvation response; SA, salicylic acid.

Rhizosphere microbiome plays an important role in channelizing many essential soil activities such as decomposition, mineralization, aggregate formation, and plant disease biocontrol, all of which are crucial to optimize citrus productivity (Lazcano et al., 2021). A better understanding of citrus associated microbiomes would impart inevitable consequences on microbial biodiversity, nutrients mobilization, supply of nutrients, nature of multi-directional associations, and soil-plant health maintenance defining the total functionality of ecosystems (Mahmud et al., 2021; Zhang et al., 2021) for multi-dimensional outcomes (Figure 1).

Citrus is an important fruit crop, known globally to contribute significantly in economic, nutritional value, and health importance due to the abundance of the vital nutrient, antioxidants, minerals, vitamins, and dietary fibers present in the juice and fresh fruits (Kreitzman et al., 2020). Above all, the distinct flavors of citrus fruits are extensively preferred and recognized throughout the world (Wang et al., 2017; Trivedi et al., 2021). Commercial citrus cultivation encounters many challenges in the field, with regard to the optimum supply of nutrients in the rhizosphere and is often exposed to untimely nutrient shortage on account of various diseases, either soil-borne or nursery plant origin. In this context, HLB is a globally dreaded disease caused by the bacteria belonging to the genus Liberibacter (Das et al., 2021). The causal agent of HLB predominantly, *Candidatus Liberibacter asiaticus* (*C*Las) is restricted to grow and colonize within phloem tissues and does not interact with other microbial flora of the citrus microbiome directly, unless they survive in the phloem of citrus (da Graca et al., 2016; Ginnan et al., 2020). Liberibacter is a systemic bacterial pathogen and causes regulatory metabolic changes in their citrus hosts, which disturbs the citrus microbiome in multiple directions. Predominantly, photoassimilates transport is impaired mainly due to phloem malfunction and reducing the release of plant-derived photosynthates at the initial phase of the Huanglongbing (HLB) infection process (Zhang et al., 2017; Trivedi et al., 2020). At the initial phase of HLB invasion, roots of citrus plants expressively decline, impeding with the loss of carbonaceous compounds from root tissues. HLB also affects the plants’ defense system, which in turn disturbs the core microbiota of the citrus rhizosphere (Zhang et al., 2017; Trivedi et al., 2021).

Apart from disturbing the core microbiome, HLB also brings about nutrient imbalances arising from damages affecting the root architecture, directly disturbing the processes associated with absorption, assimilation, transport, and utilization of nutrients and water (Johnson et al., 2014; Wang, 2020). Likewise, pathogen blocks nutrient resources, either inside infected tissues or within the rhizosphere, leading to under-supply of the number of nutrients (Dordas, 2008; da Silva et al., 2020). Elevated micronutrient fertilization is popularly utilized to enhance the growth and development of HLB-infected citrus plants (Dong et al., 2021b). Additionally, micronutrient is reported to elicit systemic acquired resistance in citrus plants acting as elicitors to reduce disease-related injuries and inhibit the development of pathogens, either by knock-down effect or by controlling gene-linked pathogenicity (Dordas, 2008; Spann and Schumann, 2010; Gottwald et al., 2012; Fones and Preston, 2013). Hence, the changes in the composition of phloem sap mainly due to under supply of different micronutrients revealed some useful insights for inactivating the development and sustenance of *C*Las in the phloem (Hijaz and Killiny, 2014; Hijaz et al., 2016; Killiny, 2016; Dong et al., 2021a). Indeed, researches have shown that zinc (Zn) can reduce bacterial infection, followed by manganese (Mn) playing an important role in the synthesis of non-structural carbohydrates, nitrogen metabolism, phenols, and phytoalexin in citrus (Srivastava et al., 2005; Datnoff et al., 2007; Bargaz et al., 2018; Ray et al., 2020). Moreover, copper (Cu) has also been applied for several years to control the phytosanitary problems triggered by micro-communities (Russell, 2005; Trivedi et al., 2021). Considering all these collectively, it is feasible that enhancing the concentration of micronutrients in plants might reduce the deleterious impact of HLB in citrus plants (Dong et al., 2021b). Despite these facts, the effect of various micronutrients supplies on *C*Las acquisition by adults and nymphs of citrus psyllids is still in a rudimentary stage of our understanding.

Considering the severity of *C*Las imparting substantial loss of plants vigor and soil health, several researchers are using microbes mediated remediation technology in the current cultivation scenario (Bora and Bora, 2020, 2021; Dong et al., 2021b). Microorganisms have been used as biocontrol/biopesticides (Mishra et al., 2018; Saikia et al., 2021) as well as biofertilizers biostimulants (Ngullie et al., 2015; Srivastava et al., 2015, 2021). However, the researchers in the current past-HLB scenario have only achieved partial success toward effective control of HLB due to the unculturable nature of *C*Las *in vitro*. Recently, the research report published by the Japanese team stated that the putative causal agent of citrus greening (*C*Las) could be successfully cultured in the system of co-culture with *Pseudomonadaceae, Comamonadaceae, Microbacteriaceae*, and *Flavobacteriaceae* in a modified culture medium with the addition of essential vitamins and nutrients (Killiny-Mansour, 2019). Though, it remained a mystery about culturing of HLB pathogen in a monoculture system, restricting researchers to provide sustainable protection against HLB to date. Despite all these fruitful efforts, microbiome-mediated remedies of HLB and soil health management are on a continuous upsurge with serious concerns, however, it still remains unexplored. Nevertheless, modern biotechnological breakthroughs have paved the way for use of microbiomes and their secondary metabolic products addressing soil-plant health-related issues of citrus.

The proposed review outlines and discusses the recent advancements made toward the citrus microbiome, their core taxonomic, and functional traits involved in citrus protection, in addition to the revitalization of citrus plants through soil health. Correspondingly, we attempted to summarize how the knowledge derived from multi-omics technologies has provided a robust understanding of the structure and functions of citrus-associated microbiomes. Various modes and approaches using microbiomes to improve the performance of citrus plants were critically reviewed by the authors.

## Hub and Core Microbiome of Citrus

Numerous high-throughput sequencing (HTS) based studies have suggested that the core microbiome is a cluster of micro-communities that typically reside inside the host’s microbiome (Compant et al., 2019; Simonin et al., 2020; Trivedi et al., 2021). The core and hub microbiome of citrus compartments (rhizosphere, rhizoplane, and phyllosphere) have been systematically investigated from various angles (Blaustein et al., 2017; Xu J. et al., 2018; Trivedi et al., 2021). Surprisingly, significant overlaps were recorded within different members of the core microbiome through several interactions of either the same plant species or phylogenetically different. Such an imperative overlap implies a possibility that certain specific groups of bacteria have a preferential association with plants for a longer period of time (Simonin et al., 2020; Trivedi et al., 2021), while others failed to survive. Several prominent researchers have stated that a group of micro-communities exhibit their stable and strong association with specific hosts across geographically different habitats (Blaustein et al., 2017; Hamonts et al., 2018; Xu L. et al., 2018). These microorganisms are not only persistent and predominant but occur plentifully in nature.

Various members of the core microbiome are hypothesized to perform a crucial role in shaping the assembly of plant-associated microbiota, besides acting as a key regulator of plant growth and development (Toju et al., 2018; Zhang et al., 2021). Different parts of citrus plants comprise distinct members of core microbiomes (Xu J. et al., 2018; Ginnan et al., 2020; Zhang et al., 2021). Precisely, different compartments of citrus encompass various bacterial population in core microbiomes consist of diverse genera including *Pseudomonas, Sphingobium, Chittinophaga, Agrobacterium, Steroidobacter, Mesorhizobium, Dokdonella, Cupriavidus, Novosphingobium, Rhizobium, Hylemonella, PhenylobacteriumRoseateles, Niastella, Devosia, Halomonas, Rhodoplanes, Sphingomonas, Streptomyces*, and *Bacillus*. Numerous genera of microbes are associated with these core microbiota, identified as beneficial microorganisms for the plant in other systems. These beneficial microbial members might benefit in regulating root development, upholding hormonal balance, facilitating mobilization and acquisition of nutrients, and suppressing the disease expression in the host plants (Fravel et al., 2003; Verbon and Liberman, 2016; Lemanceau et al., 2017). Till now, the core microbiome is well-defined on the basis of taxonomy as well as functional features that derive microbiome-plant associations such as colonization, signaling, and competition.

As greater importance of functional attributes of plant-associated microbiota, an emphasis is employed on defining the “central functional microbiome” of plants at various scales (Lemanceau et al., 2017; Zhang et al., 2021). Previous research revealed the core functional attributes of the citrus rhizosphere microbiome, leaving endosphere microbiomes due to complications of extracting the DNA and RNA from the microbiota of host plants. The microbiome’s core functional attributes of the citrus rhizosphere are enhanced for microbial features that facilitate microbe-microbe or microbe-plant interactions, resulting in an elevated acquisition of nutrients and plant-soil health (Xu J. et al., 2018; Trivedi et al., 2021; Zhang et al., 2021).

The citrus rhizosphere microbiome contains a large number of transporter genes responsible for phosphotransferase systems, ATP binding, and metabolite movement, which regulate the variety of plant-derived nutrients. These transporters are likely to allow fine-tuning between the microbial growth and root exudates, facilitating selective rhizosphere microbiome requirement from the bulk soil (Trivedi et al., 2020, 2021; Zhang et al., 2021). A positive selection of attributes (bacterial secretion systems and other outer surface proteins), known to interact directly with plant host, suggest that the immune response of plants plays a pivotal role in the composition of various microbial niches (Hacquard et al., 2017). Considering the importance of microbes in maintaining plant and soil health, there is every possibility to realign these microbes targeted to new crop production and management strategies. Hence, their identification, functionalities, and modes of performance in challenging environment are still a must, to be exploited.

Google scholar based bibliometric analysis of subject using four key words (“citrus” and “metagenomics,” “metagenome” and “HLB,” “metagenome” and “soil health,” “nutrients” and “HLB”) during 1900–2021 showed as an upsurge in systematic research on proposal theme, only after 2010. Whereas prior to the year 2000, hardly any research work on these issues could gather any news in the research arena. Of 4,739 publications, a maximum of 2,239 publications dealing with nutrients and HLB followed by 1,135–1,152 publications covering the issues like citrus metagenome or metagenome and soil health, and only 213 publications touching metagenome and HHLB, vividly showcase the temporal shifts in research interest worldwide. The rise in publications after 2010 was accountable for increased access to technology at affordable prices.

## Multiomics for Decoding Plant Microbiomes

The functionality of the citrus microbiome, where different microbial communities keep interacting with plants is now more precisely decoded through HTS sequencing and metagenomics (metatranscriptomics and metaproteomics).

### Amplicon Sequencing

HTS-based marker gene tags (iTAG) targeting taxonomic as well as functional genes are employed for profiling the composition, organization, and spatial distribution of micro-communities. With the help of iTAG sequencing, studies on microbiome residing inside or on the members of crops such as rice (Edwards et al., 2015), millet (Jin et al., 2017), sugarcane (Hamonts et al., 2018), wheat (Donn et al., 2015), corn (Walters et al., 2018), and pea (Tkacz et al., 2020), including citrus (Xu J. et al., 2018) have revealed some astonishing success in plant health management.

### Whole Genome Shotgun Sequencing

Shotgun sequencing is another approach that provides information on total DNA and identifies the genomic features revealing plant colonization or plant-microbial associations (Ofek-Lalzar et al., 2014; Bulgarelli et al., 2015; Xu J. et al., 2018). The signs of positive selection of several microbial traits such as bacterial T3SS, cellular mobility, utilization of carbon compounds, stress response provide evidence of plant-microbe co-evolution in the rhizosphere, predicting an innate immune system of plants (Sessitsch et al., 2012; Bulgarelli et al., 2015; Xu L. et al., 2018). A comprehensive analysis of the structural and functional composition of citrus rhizosphere microbiome employing amplicon and deep shotgun sequencing from different bio-geographical regions of six continents revealed the composition of core citrus rhizosphere microbiome comprise of *Agrobacterium*, *Bradyrhizobium*, *Burkholderia*, *Cellvibrio*, *Cupriavidus*, *Mesorhizobium*, *Paraburkholderia*, *Pseudomonas*, *Rhizobium*, *Sphingomonas*, *Variovorax*, and to harness the power of the microbiome for improving plant health and fruit yield (Bulgarelli et al., 2015; Xu J. et al., 2018).

Genomes are the integral component that provides biological information about an organism. Recent advances in genome curation allow the generation of complete metagenome-assembled genomes from very complex systems of soil and sediments (Chen et al., 2020), providing more detailed information about the functional and evolutionary aspects of plant-associated microbiomes. Integrating metagenomics with other high-throughput approaches such as metatranscriptomics, metaproteomics is better equipped to understand and assess the microbial interactions and the expression of their potential functional traits *in situ* or *ex situ* (Table 1). Metatranscriptomics as a high-throughput sequencing approach has a significant challenge of requiring enriched mRNAs, to assess the entire microbiome, without prior selection of taxonomic groups. A combined metagenomic and metatranscriptomic profiling proved to be highly effective in examining genetic potential, gene expression patterns of plant-associated micro-communities, and transcriptional profiling of host plants under stress conditions (Zolti et al., 2019).

**TABLE 1:** Metagenome genes and their functions involved in plant growth.

S. No	Gene(s)/Metabolite(s)	Functions	References
1.	MorA	Acts as a phosphodiesterase to inhibit biofilm formation	Liu et al., 2018
2.	Toxin genes	Synthesis of toxins, 2,4-diacetylphloroglucinol (*phlA)*, pyrrolnitrin (*prnA)*, hydrogen cyanide (*hcnA)*, and pyoluteorin (*pltA)*	Jousset et al., 2010
3.	N-cycling genes	Nitrogen fixing (*nifH*), ammonia oxidizing (*amoA*), denitrifying (*nirK* and *nirS*), nitrous oxide reducing (*nosZ*), and organic nitrogen decomposing (*chiA*)	Ouyang et al., 2018; Yu et al., 2019
4.	Hydrolytic enzymes	Role in penetration through the host cuticle in entomopathogenic fungi (subtilisin protease Pr1A)	Pava-Ripoll et al., 2011
5.	Non-specific acid phosphatase (ACP)	Mineralization of soil organic P and for the improvement of soil P availability (*PhoC)*	Zheng et al., 2019
6.	Secondary metabolite 2,4-diacetylphloroglucinol	Act as a signal on *Azospirillum* PGPR and enhance the phytostimulation effects of the latter	Combes-Meynet et al., 2011
7.	Polyketide synthase (PKS) and non-ribosomal peptide synthetase enzyme complexes	Synthesis of antimicrobials, siderophores, or toxins (NRPS/PKS genes)	Aleti et al., 2017
8.	Type six secretion system (T6SS)	Bactericidal activity implicated in bacterial killing and colonization within the rhizosphere	Durán et al., 2021

Insights of molecular phenotypes from micro-communities present in the rhizosphere (Moretti et al., 2012; Bona et al., 2019) and phyllosphere (Delmotte et al., 2009) of plants are effectively obtained with the help of metaproteomic analyses, since protein expression was most active in *Streptomyces*, *Bacillus*, *Bradyrhizobium*, and *Pseudomonas*.

Plant diseases are diagnosed by metabolomic approaches, but less applied to microbiome studies (Adeniji et al., 2020). Previous reports have shown that the microbiome associated with the rhizosphere brings changes in the metabolome of the phyllosphere, linked to different feeding behavior of insects (Badri et al., 2013). Changes in the root metabolome represent specific microbial communities that might alter the performance of a plant and the interactions of plant with herbivores in the next generation (Hu et al., 2018; Huang et al., 2019). Small molecules such as strigolactones and benzoxazinoids are detected and quantified by using metabolome information (Leach et al., 2017; Trivedi et al., 2020).

**FIGURE 2:**
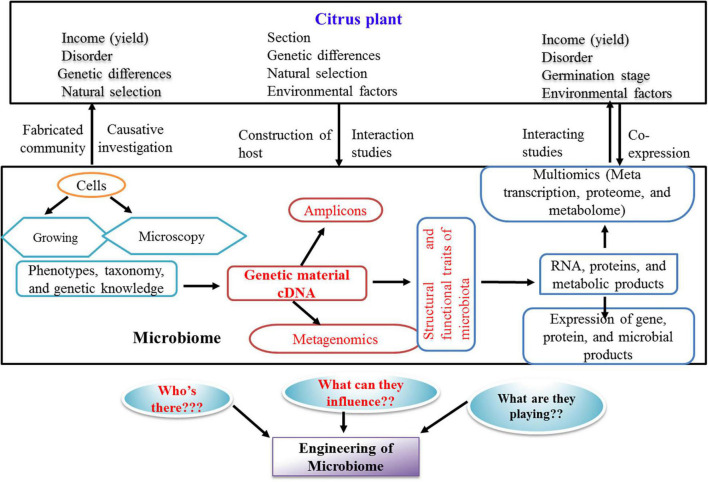
Prospective methods and questions for the research of citrus microbiome in current scenario. The present state of investigation about the citrus microbiome is highlighted in red color.

Plant-associated microbes are useful for establishing large-scale culture collections to be subjected to comparative genomics identifying homologs of proven bacterial genes, involved in colonization, pathogenesis, or nutrient supply to the plants (Finkel et al., 2017; Levy et al., 2018). The cultured members enable validate candidate genes by molecular approaches such as mutagenic and bioreporter expression systems (Cole et al., 2017; Pini et al., 2017). In order to improve the culturing of previously uncultured microbes, single-cell amplified and metagenome-assembled genomes integrated with genome-based metabolic constructions are used for the formulation of specific media recipes (Kwak and Park, 2018). Summarized studies (Figure 2) have highlighted the important steps involved in investigations on the role of citrus microbiome influencing both soil health and plant health.

## Microbiome in Soil Health of Citrus

The microbiome plays a significant role as a key driver to sustain fertility as well as the health of soil (Srivastava et al., 2021). The soil micro-communities frequently help the plant to acquire macro and micronutrients by solubilizing and mobilizing them in addition to a breakdown of organic matter through various complex mechanisms to release the immobilized nutrients (Srivastava and Malhotra, 2017). Particularly, micro-communities also use soil C and N for their growth and energy responses (Wild et al., 2014; Tian et al., 2018; Dong et al., 2021a).

Declining rock phosphate deposits, energy-intensive N-fertilizer manufacture, and emerging adverse environmental issues due to unscientific use of inorganic fertilizers have sparked interest in developing alternative strategies for plant nutrition to improve better sustainability (Jacoby et al., 2017; Dong et al., 2021b). Microbes-mediated nutrient conversion is a crucial driver for the growth of the plant, which could be the rate-determining step in the functioning of an agroecosystem (Schimel and Bennett, 2004; Dong et al., 2021a). Microbiome-plant interactions are driven by fine adjustment between the genetic response of the host and activities of related microbiomes to enable uptake of nutrients (Srivastava and Singh, 2006). For instance, plants have adaptive phosphate starvation responses (PSRs) operating in the presence of their linked microbiome to upraise orthophosphate (Pi) use efficiency in the soil environment (Castrillo et al., 2017). The structural composition of plant-associated microbiome is influenced by suppression of microbial-driven plant immune system, predominantly regulated through genetic linkage of PSR signaling system (Castrillo et al., 2017; Finkel et al., 2019). Activation of microbiome-mediated PSR was proven in response to low Pi circumstances with the help of 35-member SynCom. Finkel et al. (2019) applied 185-member SynComs through an extensive array of P concentrations in Pi-stressed situations to establish the selective recruitment of dormant opportunistic competitors impairing P-starvation.

Nitrogen (N) is an essential element for the synthesis of chlorophyll, nucleic acid, amino acids, and the energy transfer molecule adenosine triphosphate (ATP) for the plants (Werner and Newton, 2005). In the soil, one of the main N sources is organic N, utilized (mineralization) by the soil microbiome to make it easily available to plants. Another macronutrient Potassium (K), a critical inorganic cation found in the cytoplasm is involved in photosynthesis, protein synthesis, and a variety of other primary metabolic activities. The microbiome can enhance the availability of K through various modes of action, including solubilization, acidolysis, and chelation for the multiple benefits of host plants (Srivastava, 2013; Srivastava and Singh, 2006).

**FIGURE 3:**
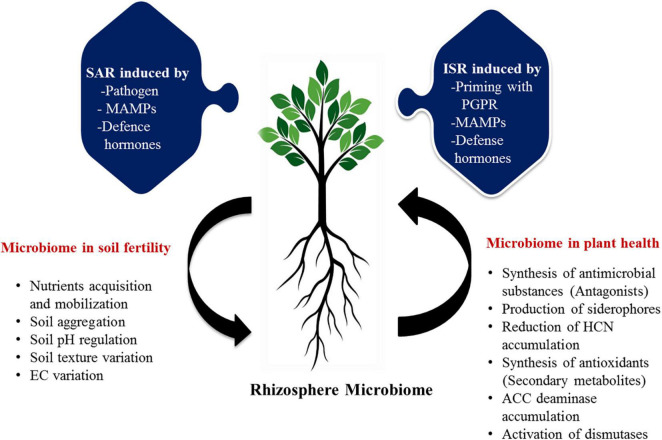
The proposed possible roles of rhizosphere microbiome in soil and plant health of citrus. Functional traits of microbiota in the management of soil and plant health are represented in various mode of action. ISR, induced systemic resistance; SAR, systemic acquired resistance; PGPR, plant growth promoting rhizobacteria; MAMPs, microbe-associated molecular pattern; EC, electrical conductivity; HCN, hydrogen cyanide; ACC, 1-aminocyclopropane-1-carboxylic acid.

Micronutrients also play a pivotal role in sustaining soil health and ecosystem functioning (Srivastava and Singh, 2005). Numerous micronutrients comprising Zinc (Zn), Iron (Fe), Manganese (Mn), Molybdenum (Mo), and Boron (B) are essentially required for plant growth and development of citrus as they are significantly involved in photosynthesis, water oxidation, respiration, and protection against a variety of oxidative stress damages (Srivastava and Singh, 2006; Srivastava and Malhotra, 2017). Despite these, the above micronutrients are not easily available to plants as they are by and large observed in immobilized and complex forms, inaccessible to plants directly (Dong et al., 2021a). Soil microbiome performs enormously to solubilize, oxidize, and mobilize these nutrient elements by synthesizing a large number of enzymes, chelators, and organic acids. There are many members of citrus-based bacterial microbiome (*Bacillus, Burkholderia, Pseudomonas, Sphingobium, Chittinophaga, Agrobacterium, Steroidobacter, Mesorhizobium, Dokdonella, Cupriavidus, Novosphingobium, Rhizobium, Hylemonella, Phenylobacterium, Roseateles, Niastella, Devosia, Halomonas, Rhodoplanes, Sphingomonas*, and *Enterobacter*), actively involved in the solubilization and availability of micronutrients in the soil (Verbon and Liberman, 2016; Lemanceau et al., 2017; Lee et al., 2019; Zhang et al., 2021). These microbes oxidize different micronutrients by using different modes of action and make them available directly to the citrus plants. The actual and predicted functions of the microbiome in the sustainable maintenance of soil and plant health could be visualized (Figure 3).

Interestingly, recent research stated that the microbiome colonizes the soil and forms biofilm via the synthesis of exopolysaccharides, eDNA, eProteins, and alginate (Ansari and Ahmad, 2019). Rhizosphere colonization by the microbiomes enhances soil aggregation by rearranging primary soil particles around the soil organic carbon, providing the required protection against mechanical hindrances. Microbiome-soil colonization and biofilm formation maintain the microbial diversity richness and soil fertility in an ecofriendly manner (Ansari and Ahmad, 2019; Ansari et al., 2019; Trivedi et al., 2021). However, the actual role and mechanisms of microbiome-soil colonization are yet to be explored to enhance our understanding to improve soil health with a more sustainable impact on plant health.

Soil microbiome biomass either live or dead, also play a crucial role in soil health by balancing the nutrient pool in form of microbial turnover determined by cell death and cell production of microbes (Kastner et al., 2021). Microbial dead cells adhere either to the soil particles and enter the soil organic carbon (SOC) pool or are metabolized by micro-communities (Hagerty et al., 2014; Dong et al., 2021b). Soil microbes mediate the cycling of carbon and other key nutrients, and therefore, a key goal of a microbes-to-ecosystem model is to identify exact relations among biogeochemical cycling and microbial composition or abundance. Subsequently, enhanced microbial turnover can improve respiration per unit of soil microbial biomass carbon (Kivlin and Hawkes, 2020). Yet, in the context of the citrus microbiome, the possible linkages between soil micro-communities turnover and their linked functions pose a greater challenge to researchers in the current era.

## Microbiome in Citrus Plant Health

In a plant microbiome, microbes interact with the plants and also with each other, synergistically promoting plant growth and protecting them against various diseases. Pathogenic microbes when introduced into the host act according to the host environment, and microbes present in or around the host (Trivedi et al., 2016; Bora et al., 2021). Thus, the plant health and disease severity depend on the level of interactions between the host plant, surrounding microbes, invading pathogens, and the prevalent environmental conditions. The plant microbial community, therefore, plays a crucial role in promoting plant growth, imparting tolerance against abiotic stresses, and lowering the impact of pathogenic invasion into the host plants. In a microbiome, some microbial members may suppress the pathogens, by pathogenicity of competing for the nutrients or by stimulating plant defense mechanisms to neutralize the invading pathogens (Trivedi et al., 2012; Ginnan et al., 2020). While some members of the microbiome may become the targets of pathogen attack (with the production of virulent factors) leading to a change in the structure of the microbiome, eventually causing a rapid decline in the health of the host plants. Consequently, studies have been performed to elucidate the possible interactions between invading pathogens and the residents of the microbiome showing promising results in relation to host fitness (Kwak et al., 2018).

Various pathogens are well known for weakening citrus health by perturbations in the host’s physiology. Citrus crops are attacked by several groups of pathogens including viruses, viroids, bacteria, oomycetes, and fungi, inflicting severe economic loss in all the citrus growing regions around the world (Datnoff et al., 2007; Busby et al., 2017). Since changes in community structure due to pathogenic infection lead to plant health decline, studies on the interaction between pathogen and the native microbiome are attracting renewed interest, of late. A diagrammatic roadmap of the regulatory mechanisms of the host’s microbiome in plant homeostasis and health management at the cellular level is developed (Figure 1) for a better understanding of these issues.

Several research works have been carried out showing the response of pathogen invasion on the status of the citrus microbiome (Table 2). Studies revealed the effect of infection caused by HLB, canker, root rot, melanose, variegated chlorosis, and pathogenic nematode on the citrus microbiome. Resultantly, the microbial communities were realigned due to infection in host leaves, stem, roots, rhizosphere, and rhizoplane (Blaustein et al., 2017; Ginnan et al., 2020). Significant changes were noticed in the endophytic microbial community due to citrus variegated chlorosis (CVC) caused by *Xyllelafastidiosa* and CVC stimulated by endophytic *Mehthylobacteriumextorquens* (Table 2). The root-feeding nematode (*Tylenchulussemipenetrans*) infected the citrus roots causing shifts in the rhizosphere microbiome, leading to an increase in the population of *Bacillus megaterium* and *Burkholderia cepacia* having a deleterious effect on the growth of root rot pathogen, *Phytophthoranicotianae* (El-Borai et al., 2003). Some bacterial genera like *Bacillus, Methylobacterium, Halomonas*, etc. were observed abundantly in citrus leaves and fruits following the infection with canker and melanosis diseases (Li et al., 2021).

**TABLE 2:** Effect of important diseases on various changes in the citrus microbiome.

Disease (Pathogen)	Microbial niche	Associated genera/phyla reported	References
HLB (*C*Las)	Rhizoplane	Reduced abundance of *Bradyrhizobium* and *Burkholderia* with reduced functional attributes.	Zhang et al., 2017
HLB (*C*Las)	Rhizosphere and endosphere	Increase in population of *Amycolatopsis*, *Sphingopyxis*, *Chryseobacterium*, *Flavobacterium*, *Ralstonia*, *Stenotrophomonas*, *Duganella*, and *Streptacidiphilus*, in addition to decrease in *Rhizobium.*	Li et al., 2021
HLB (*C*Las)	Endosphere	Reduced relative abundance of the fungal phylum *Glomeromycota* and increased abundance of fungal genera, *Fusarium* and *Gibberella*	Ginnan et al., 2020
HLB (*C*Las)	Endosphere	Enriched populations of *Exophiala* sp. and *Lactobacillus* spp. during the early stages of HLB significantly reduced in highly symptomatic trees	Ginnan et al., 2020
HLB (*C*Las)	Phyllosphere	Enriched abundance of *Aureobasidium* sp. and *Methylobacterium* spp. in early to moderate stages of HLB progression	Ginnan et al., 2020
HLB (*C*Las)	Phyllosphere	Decrease in the diversity of alpha bacteria with HLB progression. Others such as *Alphaproteobacteria* (*Methylobacterium*, *Sphingomonas*, and *Methylocystaceae*) were higher in leaves of asymptomatic trees.	Blaustein et al., 2017
HLB (*C*Las)	Endosphere	Dominant core genera associated with roots in asymptomatic trees were *Kaistobacter* and unclassified genera as *Bradyrhizobiaceae* and *Xanthomonadaceae. While other core genera Steroidobacter* and unclassified genera as *Comamonadaceae, Hyphomicrobiaceae*, MND1, IS-44, and *Rhizobiales were greater in HLB- symptomatic trees.*	Blaustein et al., 2017
Citrus variegated chlorosis (*Xylella* *fastidiosa*)	Endosphere	Presence of endophytic *Mehthylobacterium extorquens.*	Araujo et al., 2002; Lacava et al., 2004
Nematode (*Tylenchulus semipenetrans*)	Endosphere	Increase in abundance of *Bacillus megaterium* and *Burkholderia cepacia.*	El-Borai et al., 2003
Root rot and gummosis (*Phytophthora nicotianae*)	Endosphere	Enrichment of *Bacillus megaterium* and *Burkholderia cepacia* having negative effect on growth of root rot pathogen	El-Borai et al., 2003
Melanose (*Diaporthe citri*)	Phyllosphere	Bacterial diversity belonged to two functional categories: plant growth promoting (*Bacillus Methylobacterium*, and *Sphingomonas*) and plant metabolism-related (*Methylocella* and *Zymobacter*).	Li et al., 2020
Canker (*Xanthomonas citri* subsp. *citri*)	Phyllosphere and carposphere	The abundance of *Bacillus* in leaves and fruits samples, besides *Stenotrophomonas*, *Halomonas*, *Shewanella*, and *Brevundimona*	Li et al., 2021

## Citrus Microbiome and Incurring Huanglongbing: A Case Study

### Huanglongbing Bacterium and Its Interaction With Citrus Host

HLB a citrus greening disease caused by a non-cultivable, and gram-negative α proteo-bacteria, *Candidatus* Liberibacter spp. *Ca. L. asiaticus* (*C*Las), is considered the most pathogenic amongst all the Liberibacter species reported until now (Das et al., 2019). This bacterium infects the most active vascular tissue of the tree, phloem, and is harbored by the insect vector Asian citrus psyllid (ACP). HLB is known as the most destructive disease in citrus as of now, incurring enormous citrus production losses the world over (Bové, 2006; Gottwald, 2010; Das et al., 2019). The mineral deficiency-like symptoms are closely associated and often confuse the researchers; this the disease is correctly diagnosed through PCR-based techniques (Das, 2004, 2009; Das et al., 2014).

It is very crucial to understand and identify the responses of host plants involved during HLB disease development to evolve systematic disease management practices. Transcription and protein expression studies have revealed the occurrence of various innate immunity components, activated by *Ca.* Liberibacter species (Kim et al., 2009; Aritua et al., 2013; Nwugo et al., 2013). It has been shown that 10% of the genes with changed expression patterns during post *C*Las infection were connected to plant defense and possible stress mechanisms. The anatomical analyses further indicated that *C*Las infection causes phloem disruption, sucrose accumulation, and plugged sieve pores (Kim et al., 2009). The deposition of starch in HLB-affected leaves was observed due to the up-regulation of three key starch biosynthetic genes. Hence, HLB-associated phloem blockage resulted due to starch-packed sieve pores rather than *C*Las bacterial aggregates. This work highlights the role of *C*Las in altering the host gene expression in the development of HLB symptoms (Kim et al., 2009; Dong et al., 2021a).

Another study comparing gene expression of stems and roots of healthy vs. citrus infected with *C*Las by employing microarray assays (Aritua et al., 2013), showed an alteration in expression of 988 genes, of which 885 were in stems and 111 in roots. Of these, 551 and 56 genes were up-regulated, while 334 and 55 genes were down-regulated in stem and root samples of HLB infected trees, respectively, compared to healthy plants. The expression of receptor-like kinases (that are the proteins localized to the surface of host cells) was elicited following *C*Las infection, showing that *C*Las PAMPs may perhaps be shifted to the cell surface during the process of infection (Mafra et al., 2013; da Graca et al., 2016).

*C*Las-infection leading to an increased level of H_2_O_2_ in leaf tissue is known to play a dual role in plants, both as a toxic byproduct of a cell and an important signal-transducing molecule elevated after HLB-infection (Maffei et al., 2006; Torres et al., 2006; Slesak et al., 2007; Liu et al., 2010). An elevation in H_2_O_2_ production results in significant damage to cells, and therefore, the antioxidant defense system detoxifies H_2_O_2_ regulated by the plants. In *C*Las infected plants, the levels of genes [superoxide dismutase (SOD), ascorbate peroxidase (APX), and catalase (CAT)] for the enzymatic antioxidants were down-regulated, resulting in depleting the plant’s ability to scavenge an increased level of H_2_O_2_ (Pitino et al., 2017). Yellowing of shoots, chlorosis and the damaged distribution of plant tissue, the typical symptoms of HLB (blotchy mottles and chlorosis) can be attributed to an increase in reactive oxygen species (ROS) production, leading to initiation of H_2_O_2_ signal and decreased activity of detoxification system in *C*Las infected plants triggered by reduced expression of the genes represented by *APX*, *CAT*, and *SOD*. In this study, the level of ATP measured through luciferase leaf disc assay was significantly high in *C*Las infected citrus leaves (Pitino et al., 2017; Dong et al., 2021a). It was also suggested that the upregulation of enzymes involved in radical ion detoxification should be considered an important mechanism for increased HLB tolerance (Killiny et al., 2016; Martinelli et al., 2016).

### Effect of Huanglongbing on Citrus Microbiome

HLB has been observed to alter the structural and functional ability of citrus endosphere, rhizosphere, or rhizoplane based on 16S rDNA clone library, PhyloChip, and GeoChip method (Sagaram et al., 2009; Trivedi et al., 2010, 2011, 2012). The metagenomic analysis revealed that 99% of the citrus root-associated microbiome was dominated by bacteria, of which *Burkholderia* and *Bradyrhizobium* were abundant on the root surface. On the other hand, the diversity associated with *Acidobacteria* and *Actinobacteria* were more diminished on rhizoplane and rhizosphere (Zhang et al., 2017), suggesting the greater activeness of rhizoplane than rhizosphere microbiome with regard to functional features such as motility, chemotaxis, secretion systems, and lipopolysaccharide (LPS) synthesis. The abundance of other rhizoplane-associated microbes such as *Variovorax* and *Bdellovibrio*known to promote plant growth under healthy conditions was reduced with *C*Las infection. Due to *C*Las infection, the functional features of the rhizoplane microbiota such as flagellar assembly, chemotaxis, LPS synthesis and transport, secretion system, and associated effectors were significantly depleted, which suggested the adverse effect of HLB on the citrus microbiome, including the host-microbiome interactions of beneficial nature (Zhang et al., 2017).

The abundance of different microbial communities characterized by Illumina sequencing of 16S rRNA genes in asymptomatic and symptomatic leaves and roots of citrus varied with the severity of HLB symptoms. A harmonious relationship between *C*Las and members of *Micromonosporaceae, Burkholderiaceae*, and *Xanthomonadaceae* was identified by network analysis of microbial communities revealing the new associations of certain bacteria and an economically important phytopathogen (Blaustein et al., 2017). The summarized studies on microbial diversity in response to the presence of HLB infection showed important shifts in the microbiome (Table 2) to be examined toward microbes-assisted plant health management.

Application of 16SrRNA sequencing and metagenomics provided a comprehensive analysis of the citrus microbiome and its response during *C*Las infection (Li et al., 2021). A total of 30 rhizosphere and 14 root bacterial genera were affected by *C*Las infection, of which 9 were plant resistance-associated bacterial genera (*Chryseobacterium*, *Amycolatopsis*, *Sphingopyxis*, *Duganella*, *Flavobacterium*, *Ralstonia*, *Streptacidiphilus*, and *Stenotrophomonas*) loaded in *C*Las-infected roots, while *Rhizobium*population depleted. The abundance of genes involved in carbohydrate metabolism, glycolysis, starch and sucrose metabolism, amino sugar, and nucleotide sugar metabolism was reduced in the citrus rhizosphere microbial community of citrus rhizosphere during the process of HLB-infection. These results were highly effective in understanding the rhizosphere responses to HLB disease and the possible development of microbial antagonists-based products against HLB (Li et al., 2021).

A metagenomic pipeline was developed for bacteriomic analysis of HLB and ACP using next-generation sequencing techniques (Huang et al., 2021). The study identified bacteria in both, citrus as a host plant and vector psyllids, which included *Buchnera*, *Bradyrhizobium*, *Burkholderia*, “*Candidatus*Carsonellaruddii,” “*Candidatus* Profftella armature,” *C*Las, *Pseudomonas, Mesorhizobium*, *Paraburkholderia*, and *Wolbachia*. Such an outcome would be highly useful in understanding HLB biology and its management (Huang et al., 2021). Profiling of endophytic microbes in HLB-infected and HLB-free leaf midribs of Shatangju mandarin, employing next generation sequencing, revealed 53 endophytic bacterial orders through 12 phyla and 24 endophytic fungal orders distributed through 2 phyla in healthy leaf midribs (Yan et al., 2021). Among endophytic bacteria, *Actinobacteria*, *Bacteroidetes, Proteobacteria*, and *Firmicutes* were observed most predominant. While amongst endophytic fungi, members of *Ascomycota* and *Basidiomycota* were predominant. In HLB-infected leaf midribs, the diversity and richness of the endophytes were severely affected due to proportionately lower concentrations of nutrients. Some bacterial endophytes were not detected in *C*Las infected leaves namely, *Methylotenera* (known to be involved in plant growth promotion), *Lysobacteris* (known to suppress damping-off disease), and *Methylobacillus* (produce biologically active gibberellic acid GA_3_). While *Pseudomonas protegens* known to produce 2,4diacetylphlorogucinol and pyoluteorin were detected in *C*Las infected leaves for protecting the host plants. These changes in microbial patterns in *C*Las infected citrus trees offer some useful insights into the successful management of HLB (Yan et al., 2021).

### Citrus Microbiome in Huanglongbing Mitigation

HTS techniques are quite handy in providing an in-depth knowledge of plant-associated microbiomes to deduce the cross-talk between pathogens and the host plants (Paasch and He, 2021). To develop anti-*C*Las bio inoculants for citrus holosystem, HTS-based bulk culturing and microbial identification, an *in vitro* agar diffusion inhibition bioassay was integrated with culturing pipeline for identifying the microbes having antimicrobial properties against *Liberibacter crescens*, a culturable surrogate for the non-culturable CLas associated with HLB. In this study, microbes with inhibitory activity against *C*Las were identified as *Cladosporiumcladosporioides* and *Epicoccumnigrum*(fungal species) and *Bacillus*, *Curtobacterium*, and *Pantoea* (bacterial species). Purified natural products having anti- *C*Las were also identified from the fungus, *C. cladosporioides* (Blacutt et al., 2020).

In another study, *C*Las survival was demonstrated to depend on a specific subset of *C*Las-associated microbiota. *C*Las was inhibited following the elimination of a specific subset of *C*Las associated microbiome with oxytetracycline treatment, which led to the hypothesis that survival of *C*Las is promoted by the presence of *C*Las-associated microbes, thereby, aiding in accelerating the HLB menace (Blaustein et al., 2017). Further, incubation of *C*Las strain Ishi-1 mixed with *C*Las associated microbiota and oxytetracycline showed that the latter affected the growth of *C*Las-associated microbes, ultimately inhibiting the growth of *C*Las. Comparative analysis of 7,02,618 high quality sequences and 9,304 operational taxonomic units of bacteria generated through 16S rDNA sequences from oxytetracycline-treated and water-treated communities, revealed a significant reduction in a subset of bacteria belonging to classes, *Flavobacteria, Actinobacteria*, and *Proteobacteria* from a former set of treatment (Fujiwara et al., 2018). This study suggested that the above subset of bacteria might be responsible for the promotion of *C*Lasresponsible for expression of disease symptoms in the host.

In a recent study, the role of *Bacillus amyloliquefaciens* (strain GJ1) in protecting citrus against HLB was unfolded (Nan et al., 2021). It is now a known fact that citrus with infected HLB possesses blocked phloem with excess accumulation of starch and sugar in the leaves (Tang et al., 2018), damaging the chloroplast functions and affecting the transport of photosynthetic products. It was earlier reported that *B. amyloliquefaciens* reduced the infectivity of HLB (Tang et al., 2018) through an enhanced rate of photosynthesis and reduced accumulation of starch, eventually clearing the blockage of phloem. The plant resistance gene profiling in *B. amyloliquefaciens* treated plants showed the expression of genes *WRKY22* and *GST1*, known to stimulate host immunity and up-regulated significantly. The ROS accumulation was also observed significantly higher in treated citrus leaves as compared to untreated leaves, up-regulating the defense-related genes. These results disclosed that *B. amyloliquefaciens* could be an effective antagonist against HLB (Nan et al., 2021).

## Conclusion

The previous research findings have though contributed a better interpretation about citrus microbiome comprising taxonomic composition and functional traits across different niches, locales, and disease situations. However, the use of citrus microbiome in maintaining soil health and plant disease management is still in an infancy stage of understanding. By exploiting soil and plant health regulations through microbiome engineering, we need to develop a level of comprehension about the citrus microbiome and its total functionality. We streamlined some important gaps needing systematic readdressed including (i) systematic determination of structural makeup, linking citrus plant traits with microbial traits in a mutually beneficial domain and genetic potential of the citrus-associated microbiome to unlock multilateral interspecies interactions and metabolic features; (ii) ensuring efficacy, accuracy, and reproducibility in experimental vs. natural environments to move beyond cause-and-effect relationship; (iii) bioprospecting of novel growth-promoting and antagonistic microorganisms (and/or their biomolecules) isolated from citrus-associated microbiomes; (iv) development and prediction of host genotype, microbiome genotype, associated environment, and controlling interactions to modify microbial formulations; and (v) advancement and implementation of standardized processes for collating consistent and well-annotated metadata analysis through HTS and metagenomics. These suggested gaps are major challenges to be explored to maintain and protect soil and plant health in a cost-effective manner, where the citrus microbiome would be a service provider.

Broader use of multi-omics approaches, network analysis, microbial consortia, genome editing, artificial intelligence, and high throughput culturing would greatly elevate our understanding of the citrus microbiome and further unravel the microbiome’s potential toward improving citrus health via better rhizosphere ecological interventions. The full potential of citrus-associated functional genes and secondary metabolites is barely explored as yet and therefore warrants continued experimentation.

## Author Contributions

AS, AD, PJ, and PB developed the concept of review. AS, AD, FA, and RB compiled the literature. FA and RB designed the manuscript. AS, AD, PJ, PB, FA, and RB involved in writing and editing the manuscript in its present form. All authors contributed during the preparation of this study.

## Conflict of Interest

The authors declare that the research was conducted in the absence of any commercial or financial relationships that could be construed as a potential conflict of interest.

## Publisher’s Note

All claims expressed in this article are solely those of the authors and do not necessarily represent those of their affiliated organizations, or those of the publisher, the editors and the reviewers. Any product that may be evaluated in this article, or claim that may be made by its manufacturer, is not guaranteed or endorsed by the publisher.
